# Ag-Sensitized Yb^3+^ Emission in Glass-Ceramics

**DOI:** 10.3390/mi9080380

**Published:** 2018-07-31

**Authors:** Francesco Enrichi, Elti Cattaruzza, Maurizio Ferrari, Francesco Gonella, Riccardo Ottini, Pietro Riello, Giancarlo C. Righini, Trave Enrico, Alberto Vomiero, Lidia Zur

**Affiliations:** 1 Museo Storico della Fisica e Centro Studi e Ricerche “Enrico Fermi”, Roma 00184, Italy; maurizio.ferrari@ifn.cnr.it (M.F.); gonella@unive.it (F.G.); giancarlo.righini@centrofermi.it (G.C.R.); zur@fbk.eu (L.Z.); 2 Division of Materials Science, Department of Engineering Sciences and Mathematics, Luleå University of Technology, Luleå 97187, Sweden; alberto.vomiero@ltu.se; 3 Dipartimento di Scienze Molecolari e Nanosistemi, Università Ca’ Foscari Venezia, Mestre 30172, Venezia, Italy; cattaruz@unive.it (E.C.); ottini-r@live.it (R.O.); riellop@unive.it (P.R.); enrico.trave@unive.it (T.E.); 4 Istituto di Fotonica e Nanotecnologie del Consiglio Nazionale delle Ricerche (IFN-CNR), Laboratorio CSMFO and Fondazione Bruno Kessler (FBK) Photonics Unit, Povo 38123, Trento, Italy; 5 Istituto di Fisica Applicata Nello Carrara del Consiglio Nazionale delle Ricerche (IFAC-CNR), Sesto Fiorentino 50019, Firenze, Italy

**Keywords:** sol-gel, Ag nanoaggregates, Yb^3+^ ions, down-shifting, photonic microdevices

## Abstract

Rare earth doped materials play a very important role in the development of many photonic devices, such as optical amplifiers and lasers, frequency converters, solar concentrators, up to quantum information storage devices. Among the rare earth ions, ytterbium is certainly one of the most frequently investigated and employed. The absorption and emission properties of Yb^3+^ ions are related to transitions between the two energy levels ^2^F_7/2_ (ground state) and ^2^F_5/2_ (excited state), involving photon energies around 1.26 eV (980 nm). Therefore, Yb^3+^ cannot directly absorb UV or visible light, and it is often used in combination with other rare earth ions like Pr^3+^, Tm^3+^, and Tb^3+^, which act as energy transfer centres. Nevertheless, even in those co-doped materials, the absorption bandwidth can be limited, and the cross section is small. In this paper, we report a broadband and efficient energy transfer process between Ag dimers/multimers and Yb^3+^ ions, which results in a strong PL emission around 980 nm under UV light excitation. Silica-zirconia (70% SiO_2_-30% ZrO_2_) glass-ceramic films doped by 4 mol.% Yb^3+^ ions and an additional 5 mol.% of Na_2_O were prepared by sol-gel synthesis followed by a thermal annealing at 1000 °C. Ag introduction was then obtained by ion-exchange in a molten salt bath and the samples were subsequently annealed in air at 430 °C to induce the migration and aggregation of the metal. The structural, compositional, and optical properties were investigated, providing evidence for efficient broadband sensitization of the rare earth ions by energy transfer from Ag dimers/multimers, which could have important applications in different fields, such as PV solar cells and light-emitting near-infrared (NIR) devices.

## 1. Introduction

Rare earth ions (RE^3+^) are widely used in many optical materials and devices, mainly due to their unique spectral properties, which are related to the distribution of their electronic energy levels (spanning from UV to IR), narrow bandwidths and long lifetimes [1]. This makes them excellent candidates for many applications such as lighting [2,3], displays [4], biosensing [5,6,7], optical amplification [8], anticounterfeiting [9], and photovoltaic (PV) solar cells [10,11,12,13]. Among rare earths, Yb^3+^ ions provide light-emitting near-infrared (NIR) absorption and emission features peaked around 980 nm (1.26 eV), related to transitions between the two energy levels ^2^F_7/2_ (ground state) and ^2^F_5/2_ (excited state). Therefore, Tb^3+^ ions are often used as co-dopants, allowing the additional possibility of obtaining down-conversion splitting of one 488 nm photon into two 980 nm photons [14,15,16]. Nevertheless, even in co-doped materials, the limited excitation/absorption bandwidths and the small absorption cross sections of RE^3+^ ions are major limitations for their effective implementation and use in real devices.

In the last two decades, broadband and efficient sensitization of Er^3+^ ions by silicon [17,18,19] or silver aggregates [20,21,22,23,24,25] have been reported, showing that multimers and nanoaggregates can act as energy-transfer centres to the RE^3+^ ions. More recently, Ag sensitization was successfully observed in Tb^3+^ [26,27,28] and Tb^3+^/Yb^3+^ [29] co-doped materials. In this paper, we report the investigation of the direct interaction between Ag nanoaggregates and Yb^3+^ rare earth ions in sol-gel silica-zirconia glass-ceramic (GC) waveguides. GC films are good candidates for the realization of guided-wave optical planar devices [30]. A GC is constituted by a homogeneous dispersion of ceramic nanocrystals in a glass matrix. According to Extended X-ray Absorption Fine Structure (EXAFS) studies, RE^3+^ ions tend to be incorporated into the ceramic nanocrystals [31], providing a better spectroscopic environment for RE^3+^ ions. Zirconia, for example, has a lower maximum phonon energy than silica [32] and a higher refractive index. The combination of Ag-mediated enhancement with the advantage of the glass-ceramic material is studied, suggesting the possibility to exploit this material for more efficient optical devices. 

## 2. Experimental

Undoped and 4 mol.% Yb doped films of nominal molar composition 70% SiO_2_-30% ZrO_2_ and additional 5 mol.% Na_2_O were prepared by sol-gel technique and deposited by dip-coating. All the reagents were purchased by Sigma Aldrich (Saint Louis, MO, USA). Tetraethyl orthosilicate Si(OC_2_H_5_)_4_ (TEOS ) and zirconium propoxide Zr(OC_3_H_7_)_4_ (ZPO) were used as precursors for silica and zirconia, respectively. TEOS was dissolved in ethanol (EtOH) and hydrolized with H_2_O and HCl (TEOS:HCl:H_2_O:EtOH = 1:0.01:2:25). For sake of simplicity, we will refer to this solution as Sol:Si, and an analogous label will be used for the other solutions used in the process. For Yb co-doped samples, 4 mol.% ytterbium nitrate YbNO_3_ was added to the solution (Sol.Si-Yb) and then left stirring for 1 h. In the meanwhile, ZPO was mixed with acetylacetone (Acac) and ethanol (ZPO:Acac:EtOH = 1:0.5:50) and sodium acetate was dissolved in methanol (60 mg/ml), the two solutions will be referred to as Sol.Zr and Sol.Na, respectively. The deposition solution has been obtained by mixing Sol.Si-Yb with Sol.Zr and adding dropwise Sol.Na at room temperature. The solution has been left stirring overnight for about 16 h.

Multi-layer films have been deposited on fused silica substrates by dipping. Each layer was annealed in air at 700 °C for 3 min. A final heat treatment in air at 1000 °C for 1 h was performed after the deposition of the last single layer. The typical thickness of each single layer after heat treatment was about 40 nm, and, by adding 10 layers crack-free films, a total thickness of about 400 nm was achieved. By this thermal treatment, a controlled crystallization occurred and glass-ceramic (GC) film were produced.

Undoped and Yb-doped GC films were identified by GC0 and GC4 labels, respectively. In order to introduce silver in the films, Ag^+^↔Na^+^ ion-exchange [33] was performed by immersing the samples in a molten salt bath (1 mol.% AgNO_3_ in NaNO_3_) at 350 °C for 1 h. After the ion exchange process, the samples were identified by GC0-A and GC4-A. Finally, post-exchange heat treatments for 1 h at 430 °C in air (samples GC0-C and GC4-C) were used to investigate the possibility of migration and aggregation of the metal ions.

Compositional, structural, and optical characterization of the films were obtained by Rutherford Backscattering Spectrometry (RBS), X-Ray Diffraction (XRD, Panalytical, Almelo, the Netherlands), and Photoluminescence Spectroscopy.

RBS was carried out at the National Laboratories of Legaro (Padova, Italy) using a 2.2 MeV ^4^He^+^ beam at 160° backscattering angle in IBM geometry. RUMP code was used for the analysis of the experimental spectra [34]. The conversion from areal density (the natural unit of measurement for RBS) to film thickness is based on a molar density of the film equal to a weighted average between silica (2.00 g·cm^−3^) and zirconia (5.68 g·cm^−3^), according to the nominal stoichiometric composition of the matrix (70% SiO_2_-30% ZrO_2_), confirmed by the RBS analysis.

XRD measurements for crystal phase identification were carried out at room temperature by an X’Pert PRO diffractometer. A Cu anode equipped with a Ni filter was used as a radiation source (Kα radiation, λ = 1.54056 Å). Diffractograms were collected in Bragg-Brentano geometry using a step-by-step scan mode in the 2θ range 10°–100°, with a scanning step of 0.05° and counting time of 30 s/step. Nanocrystals’ size was determined by Line Broadening Analysis (LBA) [35], in particular, by using the Warren-Averbach method.

Photoluminescence excitation (PLE) and emission (PL) spectra in the UV-visible range were recorded by an Edinburgh Instruments (Livingston, UK) FLS980 Photoluminescence Spectrometer. A continuous-wave xenon lamp was used as the excitation source for steady-state measurements, coupled to a double-grating monochromator for wavelength selection. In particular, 280 nm excitation was used for the investigation of Ag-related PL emission. The light emitted from the sample was collected by a double-grating monochromator and recorded by a photon counting R928P Hamamatsu photomultiplier tube cooled at −20 °C. The PL emission in the NIR spectral range was instead obtained by exciting the sample with the third harmonic of a pulsed Nd:YAG laser at 355 nm. The emission was analyzed by a single grating monochromator coupled to an InGaAs photodiode and using a standard lock-in technique.

## 3. Results and Discussion

**Figure 1:**
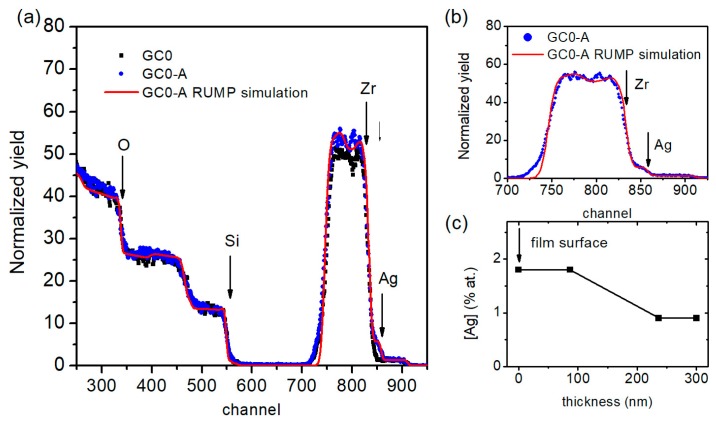
RBS spectra for undoped silica-zirconia GC samples before and after Ag-exchange (GC0 and GC0-A). The simulated GC0-A spectrum and the specific Ag contribution are also shown, resulting in Ag concentrations decreasing from 2 mol.% (at surface) to 1.5 mol.% (inner part of the film).

The elemental composition of the samples before and after Ag introduction and annealing was studied by RBS, confirming the agreement of the film composition with the nominal values for Si, Zr, and Yb. RBS analysis was also used to obtain information about the Ag concentration depth-profile after ion-exchange and annealing, revealing Ag-concentration decreasing from 2 mol.% (at surface) to 1.5 mol.% (inner part of the film) for all the samples. An example is reported in Figure 1, which presents the RBS spectra for undoped silica-zirconia GC samples before and after Ag-exchange (GC0 and GC0-A). The simulated GC0-A spectrum and the specific Ag contribution are also shown.

**Figure 2:**
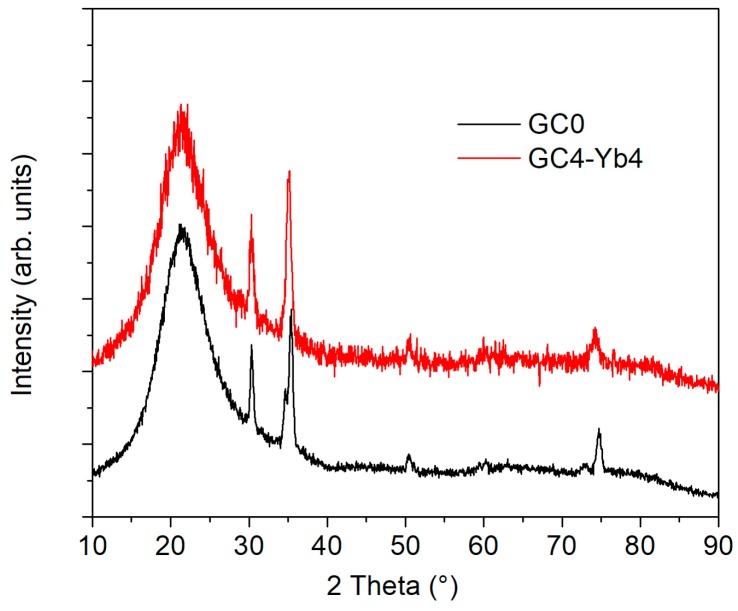
XRD comparison between silica-zirconia-soda GC samples with or without Yb co-doping. Undoped GC samples contain tetragonal-phase zirconia nanocrystals (PDF 01-080-0784; ICSD 68589), while Yb doped samples have a fluorite-type cubic-phase zirconia nanocrystals (PDF 01-078-1309; ICSD 62462), attested by the different shape of the characteristic reflection peaks, especially those at 2θ ≈ 35.5° and 2θ ≈ 75°.

The XRD analyses of the synthesized GC samples before Ag^+^↔Na^+^ ion-exchange are reported in Figure 2. It can be noted that ZrO_2_ tetragonal-phase nanocrystals were detected in the undoped GC samples (PDF 01-080-0784 50-1089; ICSD 68589), while fluorite-type cubic-phase nanocrystals were observed for Yb doped samples (PDF 01-078-1309; ICSD 62462), attested by the different shape of the characteristic reflection peaks, especially those at 2θ ≈ 35.5° and 2θ ≈ 75°. For GC0 and GC4 samples the zirconia nanocrystals’ size determined by Line Broadening Analysis (LBA) [35] was about 14 nm for tetragonal zirconia in GC0 and about 12 nm for cubic zirconia in GC4.

**Figure 3:**
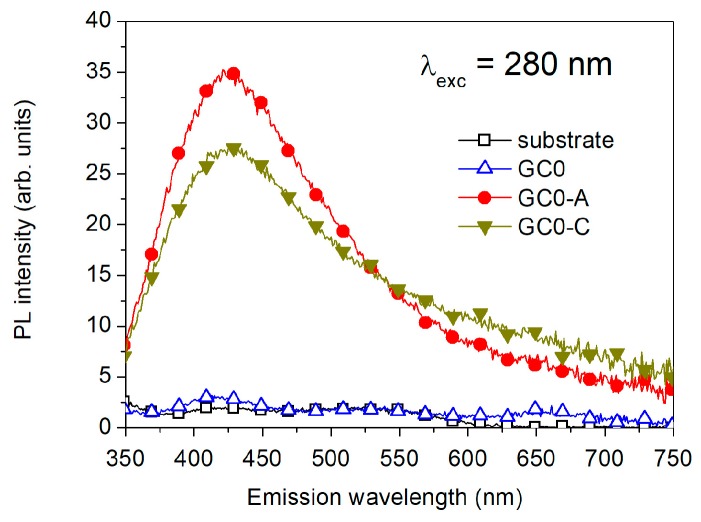
Photoluminescence emission by 280 nm excitation of undoped samples before Ag-exchange (GC0), after Ag-exchange (GC0-A), and after Ag-exchange and annealing (GC0-C). The substrate is also reported as a reference. The main contribution, peaked at 425 nm, is reasonably attributed to Ag^+^–Ag^+^ pairs. After 1 h annealing at 430 °C, the decreasing of the blue emission in favor of the red emission suggests the formation of Ag trimers and multimers.

Regarding the optical properties of the synthesized samples, it is well known that silver-doped silicate glasses exhibit broad excitation and emission bands [36,37,38,39], in relation to the emitting species. The emission around 330–370 nm is due to isolated emitting Ag^+^ ions. The emission around 430–450 nm is due to Ag^+^–Ag^+^ pairs. The emission around 550–650 nm is due to the formation of (Ag_3_)^2+^ trimers or small multimers. These bands are excited by UV and near-UV illumination. The PL emission of undoped GC samples is reported in Figure 3 before an Ag-exchange (GC0), after Ag-exchange (GC0-A), and after Ag-exchange and 1 h annealing at 430 °C (GC0-C). The curves have been obtained by 280 nm excitation. The main contribution, peaked at 425 nm, can be reasonably attributed to Ag^+^–Ag^+^ pairs. After 1 h post-exchange annealing at 430 °C, the emission shape slightly changes in the following ways: the contribution of Ag^+^–Ag^+^ pairs decreases, while the emission from trimers and multimers in the red spectral region increases. Noteworthy, and in agreement with a previous work [36], the band around 350 nm of isolated emitting silver ions was not detected, indicating that the probability of isolated ions is low.

**Figure 4:**
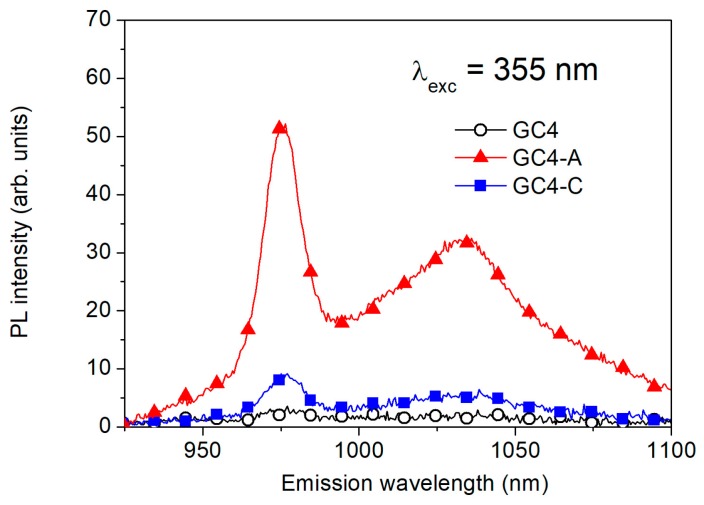
Near-infrared photoluminescence emission of Yb doped samples before Ag-exchange (GC4), after Ag-exchange (GC4-A) and after Ag-exchange and annealing (GC4-C). It can be observed that 355 nm is a very weak excitation wavelength for Yb^3+^ ions, while it results in a strong PL emission in Ag-containing samples.

The PL emission in the NIR spectral range is reported in Figure 4 for Yb^3+^ doped GC samples before Ag-exchange (GC4), after Ag-exchange (GC4-A), and after Ag-exchange and 1 h annealing at 430 °C (GC4-C). As expected, the direct excitation of Yb^3+^ by 355 nm wavelength light is very weak. However, Ag introduction by ion-exchange results in a strong enhancement of the Yb^3+^ PL emission, possibly due to Ag^+^–Ag^+^ pairs formed during the ion exchange process. During the annealing, their number decreases to form multimers or bigger aggregates, resulting in a decrease of the PL signal. Furthermore, a lower number of sensitizers means also a higher average distance between them and the Yb^3+^ ions, decreasing the efficiency of the energy-transfer process.

## 4. Conclusions

In this paper, we report the synthesis and characterization of efficient NIR emitting glass-ceramic films. In these materials, Yb^3+^ emission around 980 nm was significantly enhanced by Ag^+^↔Na^+^ ion-exchange, resulting in a strong UV absorption. The significant increase of the PL emission was attributed to energy transfer from Ag^+^-Ag^+^ pairs and multimers. Ag-sensitized Yb^3+^ doped films could have important applications as spectral downshifters for PV solar cells, converting the UV part of the solar spectrum to NIR photons around 980 nm, which can be efficiently converted to electricity by commercially available crystalline-silicon solar cells. Furthermore, the capability of sol-gel technology to synthesize high quality glass-ceramic waveguides makes these materials suitable for realizing optical devices operating in the NIR spectral range, with significant technological interest in many different fields such as optical amplifiers and lasers, frequency converters, solar concentrators, up to quantum information storage devices.

## References

[1] Liu G., Jacquier B. (2005). Spectroscopic Properties of Rare Earths in Optical Materials.

[2] Lin Y.C., Karlsson M., Bettinelli M. (2016). Inorganic phosphor materials for lighting. Top. Curr. Chem..

[3] Marin R., Sponchia G., Zucchetta E., Riello P., Enrichi F., De Portu G., Benedetti A. (2012). Photoluminescence properties of YAG:Ce^3+^,Pr^3+^ phosphors synthesized via the Pechini method for white LEDs. J. Nanopart. Res..

[4] Kim C.H., Kwon I.E., Park C.H., Hwang Y.J., Bae H.S., Yu B.Y., Pyun C.H., Hong G.Y. (2000). Phosphors for plasma display panels. J. Alloys Comp..

[5] Chen X., Liu Y., Tu D. (2014). Lanthanide-Doped Luminescent Nanomaterials: From Fundamentals to Bioapplications.

[6] Enrichi F., Riccò R., Meneghello A., Pierobon R., Cretaio E., Marinello F., Schiavuta P., Parma A., Riello P., Benedetti A. (2010). Investigation of luminescent dye-doped or rare-earth-doped monodisperse silica nanospheres for DNA microarray labelling. Opt. Mater..

[7] Enrichi F. (2008). Luminescent amino-functionalized or Erbium-doped silica spheres for biological applications. Ann. N. Y. Acad. Sci..

[8] Desurvire E. (1994). Erbium doped fiber amplifiers: principles and applications.

[9] Moretti E., Pizzol P., Fantin M., Enrichi F., Scopece P., Ocaña M., Polizzi S. (2017). Luminescent Eu-doped GdVO4 nanocrystals as optical markers for anti-counterfeiting purposes. Chem. Pap..

[10] Trupke T., Green M.A., Wurfel P. (2002). Improving solar cell efficiencies by downconversion of high-energy photons. J. Appl. Phys..

[11] Richards B.S. (2006). Enhancing the performance of silicon solar cells via the application of passive luminescence conversion layers. Sol. Energy Mater. Sol. Cells.

[12] Strumpel C., McCann M., Beaucarne G., Arkhipov V., Slaoui A., Cañizo C., Tobias I. (2007). Modifying the solar spectrum to enhance silicon solar cell efficiency—An overview of available materials. Sol. Energy Mater. Sol. Cells.

[13] Righini G.C., Boulard B., Coccetti F., Enrichi F., Ferrari M., Lukowiak A., Pelli S., Zur L., Quandt A. Light management in solar cells: Recent advances. Proceedings of the 19th International Conference on Transparent Optical Networks (ICTON).

[14] Alombert-Goget G., Armellini C., Berneschi S., Chiappini A., Chiasera A., Ferrari M., Guddala S., Moser E., Pelli S., Rao D.N. (2010). Tb^3+^/Yb^3+^ co-activated silica-hafnia glass ceramic waveguides. Opt. Mater..

[15] Bouajaj A., Belmokhtar S., Britel M.R., Armellini C., Boulard B., Belluomo F., Di Stefano A., Polizzi S., Lukowiak A., Ferrari M. (2016). Tb^3+^/Yb^3+^ codoped silica-hafnia glass and glass-ceramic waveguides to improve the efficiency of photovoltaic solar cells. Opt. Mater..

[16] Enrichi F., Armellini C., Belmokhtar S., Bouajaj A., Chiappini A., Ferrari M., Quandt A., Righini G.C., Vomiero A., Zur L. (2018). Visible to NIR downconversion process in Tb^3+^-Yb^3+^ codoped silica-hafnia glass and glass-ceramic sol-gel waveguides for solar cells. J. Lumin..

[17] Gourbilleau F., Dufour C., Levalois M., Vicens J., Rizk R., Sada C., Enrichi F., Battaglin G. (2003). Room-temperature 1.54 µm photoluminescence from Er-doped Si-rich silica layers obtained by reactive magnetron sputtering. J. Appl. Phys..

[18] Enrichi F., Mattei G., Sada C., Trave E., Pacifici D., Franzò G., Priolo F., Iacona F., Prassas M., Falconieri M. (2004). Evidence of energy transfer in an aluminosilicate glass codoped with Si nanoaggregates and Er^3+^ ions. J. Appl. Phys..

[19] Enrichi F., Mattei G., Sada C., Trave E., Pacifici D., Franzò G., Priolo F., Iacona F., Prassas M., Falconieri M. (2005). Study of the energy transfer mechanism in different glasses co-doped with Si nanoaggregates and Er^3+^ ions. Opt. Mater..

[20] Strohhöfer C., Polman A. (2002). Silver as a sensitizer for Erbium. Appl. Phys. Lett..

[21] Mazzoldi P., Padovani S., Enrichi F., Mattei G., Trave E., Guglielmi M., Martucci A., Battaglin G., Cattaruzza E., Gonella F. (2004). Sensitizing effects in Ag-Er co-doped glasses for optical amplification. SPIE Proc..

[22] Martucci A., De Nuntis M., Ribaudo A., Guglielmi M., Padovani S., Enrichi F., Mattei G., Mazzoldi P., Sada C., Trave E. (2005). Silver sensitized erbium-doped ion exchanged sol-gel waveguides. Appl. Phys. A.

[23] Mattarelli M., Montagna M., Moser E., Vishnubhatla K., Armellini C., Chiasera A., Ferrari M., Speranza G., Brenci M., Conti G.N. (2006). Silver to erbium energy transfer in phosphate glasses. J. Non-Cryst. Solids.

[24] Mattarelli M., Montagna M., Vishnubhatla K., Chiasera A., Ferrari M., Righini G.C. (2007). Mechanisms of Ag to Er energy transfer in silicate glasses: a photoluminescence study. Phys. Rev. B.

[25] Trave E., Back M., Cattaruzza E., Gonella F., Enrichi F., Cesca T., Kalinic B., Scian C., Bello V., Maurizio C. (2018). Control of silver clustering for broadband Er^3+^ luminescence sensitization in Er and Ag co-implanted silica. J. Lumin..

[26] Abbass A.E., Swart H.C., Kroon R.E. (2015). Effect of silver ions on the energy transfer from host defects to Tb ions in sol–gel silica glass. J. Lumin..

[27] Li L., Yang Y., Zhou D., Xu X., Qiu J. (2014). The influence of Ag species on spectroscopic features of Tb^3+^-activated sodium–aluminosilicate glasses via Ag^+^–Na^+^ ion exchange. J. Non-Cryst. Sol..

[28] Enrichi F., Cattaruzza E., Ferrari M., Gonella Martucci A., Ottini R., Riello P., Righini G.C., Trave E., Vomiero A., Zur L. Role of Ag multimers as broadband sensitizers in Tb3+/Yb3+ co-doped glass-ceramics. Proceedings of the SPIE Fiber Lasers and Glass Photonics: Materials through Applications.

[29] Enrichi F., Armellini C., Battaglin G., Belluomo F., Belmokhtar S., Bouajaj A., Cattaruzza E., Ferrari M., Gonella F., Lukowiak A. (2016). Silver doping of silica-hafnia waveguides containing Tb^3+^/Yb^3+^ rare earths for downconversion in PV solar cells. Opt. Mater..

[30] Ferrari M., Righini G.C. (2015). Glass-ceramic materials for guided-wave optics. Int. J. Appl. Glass Sci..

[31] Afify N.D., Dalba G., Rocca F. (2009). XRD and EXAFS studies on the structure of Er^3+^-doped SiO_2_–HfO_2_ glass-ceramic waveguides: Er^3+^-activated HfO_2_ nanocrystals. J. Phys. D Appl. Phys..

[32] Zhao X., Vanderbilt D. (2002). Phonons and lattice dielectric properties of zirconia. Phys. Rev. B.

[33] Gonella F. (2015). Silver doping of glasses. Ceram. Inter..

[34] Doolittle L.R. (1985). Algorithms for the rapid simulation of Rutherford backscattering spectra. Nucl. Instrum. Methods B.

[35] Enzo S., Polizzi S., Benedetti A. (1985). Applications of fitting techniques to the Warren-Averbach method for X-ray line broadening analysis. Z. Kristallogr. Cryst. Mater..

[36] Cattaruzza E., Caselli V.M., Mardegan M., Gonella F., Bottaro G., Quaranta A., Valotto G., Enrichi F. (2015). Ag^+^-Na^+^ ion exchanged silicate glasses for solar cells covering: down-shifting properties. Ceram. Inter..

[37] Cattaruzza E., Mardegan M., Trave E., Battaglin G., Calvelli P., Enrichi F., Gonella F. (2011). Modifications in silver-doped silicate glasses induced by ns laser beams. Appl. Surf. Sci..

[38] Borsella E., Battaglin G., Garcia M.A., Gonella F., Mazzoldi P., Polloni R., Quaranta A. (2000). Structural incorporation of silver in soda-lime glass by the ion exchange process: a photoluminescence spectroscopy study. Appl. Phys. A.

[39] Borsella E., Gonella F., Mazzoldi P., Quaranta A., Battaglin G., Polloni R. (1998). Spectroscopic investigation of silver in soda-lime glass. Chem. Phys. Lett..

