# Erratum: Enrichi, F., et al. Ag-Sensitized Yb^3+^ Emission in Glass-Ceramics. *Micromachines* 2018, *9*, 380

**DOI:** 10.3390/mi10020098

**Published:** 2019-01-29

**Authors:** Micromachines Editorial Office

**Affiliations:** MDPI AG, St. Alban-Anlage 66, 4052 Basel, Switzerland; micromachines@mdpi.com

The editorial team of the journal *Micromachines* would like to make the following change to the published paper [1]: The author’s name “Trave Enrico” should be changed to “Enrico Trave”.

We apologize for any inconvenience caused to the readers or authors by this error. The change does not affect the scientific results. The article will be updated and the original will remain on the article webpage. 

## References

[1] Enrichi F., Cattaruzza E., Ferrari M., Gonella F., Ottini R., Riello P., Righini G.C., Trave E., Vomiero A., Zur L. (2018). Ag-Sensitized Yb^3+^ Emission in Glass-Ceramics. Micromachines.

